# Intraocular Lens Modifications for Postoperative Complication Prevention: Advances in Surface Engineering, Drug Delivery, and Photo-Responsive Strategies

**DOI:** 10.3390/pharmaceutics18050616

**Published:** 2026-05-18

**Authors:** Meitong Lin, Wenlu Yu, Ke Zhang, Jiayi Wu, Xingtong Chen, Yuke Pan, Yujie Tian, Liangjia Zeng, Haorui Yuan, Xiaofei Hu, Xuhua Tan

**Affiliations:** 1 State Key Laboratory of Ophthalmology, Zhongshan Ophthalmic Center, Guangdong Provincial Key Laboratory of Ophthalmology and Visual Science, Sun Yat-sen University, Guangzhou 510060, China; txtxtxlmt@163.com (M.L.); yuwenlu1106@163.com (W.Y.); panyk@mail2.sysu.edu.cn (Y.P.); tianyujie9989@163.com (Y.T.); lucaszeng12138@163.com (L.Z.); yuanhr5@mail2.sysu.edu.cn (H.Y.); huxf23@mail2.sysu.edu.cn (X.H.); 2 Guangdong Provincial Clinical Research Center for Ocular Diseases, Guangzhou 510623, China; 3 Zhongshan School of Medicine, Sun Yat-sen University, Guangzhou 510060, China; zhangk373@mail2.sysu.edu.cn (K.Z.); wujy523@mail2.sysu.edu.cn (J.W.); chenxt277@mail2.sysu.edu.cn (X.C.)

**Keywords:** intraocular lens, posterior capsular opacification, endophthalmitis, IOL modification, surface modification, drug delivery, nano-engineered drug delivery, bio-instructive delivery, photo-responsive IOL

## Abstract

Cataract remains the preeminent cause of reversible blindness globally, with cataract extraction and intraocular lens (IOL) implantation serving as the definitive surgical intervention. Nevertheless, its long-term efficacy is undermined by formidable postoperative complications, specifically posterior capsule opacification (PCO) and endophthalmitis, which necessitate effective prophylactic strategies. IOL modification has emerged as a pivotal paradigm to effectively mitigate these complications. Current approaches encompass surface modification, drug delivery IOLs, and photo-responsive IOLs. Driven by the rapid interdisciplinary convergence of materials science, ophthalmology and pharmacology, the field has also evolved to have combined modification strategies and multifunctional systems. This review provides a comprehensive overview of the recent progress in IOL modification for postoperative complication prophylaxis. By categorizing recent advancements into three major types—surface modification, drug delivery systems, and photo-responsive IOLs—we critically evaluate their mechanisms, advantages, and limitations. Furthermore, we offer strategic insights to accelerate the development of IOL modification and bridge the gap between innovation and clinical translation.

## 1. Introduction

Cataract remains the leading cause of reversible blindness worldwide, affecting an estimated 15.2 million individuals and accounting for approximately 45.4% of blindness among patients aged above 50 years in 2020 [1,2]. With global population aging, its prevalence continues to rise, imposing a substantial socioeconomic burden, as highlighted by the Global Burden of Disease Study 2019 [3,4]. Cataract extraction with intraocular lens (IOL) implantation is currently the most effective and widely adopted treatment. However, postoperative complications may compromise visual recovery and long-term surgical outcomes.

Posterior capsule opacification (PCO) is the most common complication following cataract surgery [1,5]. Although effectively treated with neodymium-doped yttrium aluminum garnet (Nd:YAG) laser capsulotomy [6,7], this intervention increases healthcare costs and the risk of complications such as elevated intraocular pressure, cystoid macular edema, and retinal detachment [8,9,10]. Moreover, laser-induced IOL damage, including pitting and optical degradation, has become a growing concern, particularly in premium IOLs [11,12]. Endophthalmitis, though relatively rare [13], represents the most devastating postoperative complication [14,15]. Once it occurs, aggressive interventions such as intravitreal antibiotics, vitrectomy, or even enucleation may be required, often resulting in poor visual prognosis [16]. Therefore, preventing both PCO and endophthalmitis remains critical for improving long-term outcomes after cataract surgery.

### 1.1. Pathogenesis, Treatment and Prevention of PCO

PCO arises from the proliferation, migration, and epithelial–mesenchymal transition (EMT) of residual lens epithelial cells (LECs), leading to poor visual outcomes and IOL instability [17]. Based on its pathogenesis, PCO is classified into fibrotic and regenerative PCO. Fibrotic PCO results from fibrosis of anterior capsule LECs, characterized by matrix deposition, contraction, and cellular transdifferentiation [18]. In contrast, regenerative PCO originates from pre-equatorial LECs and is characterized by Soemmerring’s ring and Elschnig’s pearls [19]. Regenerative PCO is more prevalent and is associated with poorer visual outcomes, although both subtypes often coexist [20,21]. PCO is considered a wound-healing response to surgical trauma [18], which may explain its high incidence. PCO development varies from weeks to years [7,22,23], highlighting the necessity of sustained prevention.

Nd:YAG laser capsulotomy remains the standard treatment to restore visual clarity [17,18]. However, an increased risk of retinal complications including macular edema and retinal detachment [10,24,25] was reported. Additionally, the Nd:YAG laser has been reported to cause alterations in IOL positioning [26], deterioration of refractive outcomes [27], rotation of toric IOLs [26], and even IOL damage [28], potentially exerting a pronounced impact on premium IOL performance. Furthermore, extra medical expenses can impact patient satisfaction. Collectively, these limitations highlight the need for improved approaches to PCO prevention.

Several methods have been explored to prevent PCO, including the use of modified IOL materials [29,30], posterior capsulorhexis [31], pharmacological agents loaded on IOLs [5,32] and capsular tension ring implantation [33]. Increasing evidence suggests that IOL biocompatibility and surface properties critically influence LEC behavior [34,35,36]. Accordingly, IOL modification has emerged as a promising approach to reduce PCO incidence and minimize the need for Nd:YAG laser treatment.

### 1.2. Pathogenesis, Treatment and Prevention of Endophthalmitis

Endophthalmitis is a rare but vision-threatening complication following cataract surgery, with reported incidences between 0.03 and 0.5% from multiple research centers in different countries [37,38,39,40]. Both bacterial and fungal pathogens can invade the eye intraoperatively or postoperatively [39], with Gram-positive bacteria being the most common causative organisms [14,16]. Despite routine antiseptic measures [16,41], complete elimination of microbial contamination is impossible. Once bacteria adhere to the IOL, they colonize and proliferate to form biofilms resistant to host immune responses and antimicrobial therapy [42,43]. In addition to acute inflammation, biofilm can induce long-term and irreversible damage to intraocular implants and tissues, leading to permanent visual impairment [44,45].

Current treatments rely on intravitreal antibiotics, with systemic antibiotics or vitrectomy when necessary [14,38]. However, poor outcomes and extra costs call for prevention strategies. Prevention strategies include the perioperative use of antibiotic eye drops and intracameral antibiotic injections [46,47]. However, the antibiotic efficacy is limited by unsatisfactory bioavailability [48,49], toxicity [47] and complications such as hemorrhagic occlusive retinal vasculitis [50,51] and cefuroxime-induced toxic retinopathy [52,53]. Furthermore, widespread antibiotic use contributes to an increasing prevalence of multidrug-resistant bacteria, posing a significant threat to medical safety [15,16,54]. Enhancing the intrinsic antibacterial properties of IOLs therefore represents an attractive alternative for reducing antibiotic dependence and preventing endophthalmitis.

### 1.3. Progress and Limitation in IOL Material Modification

**Figure 1:**
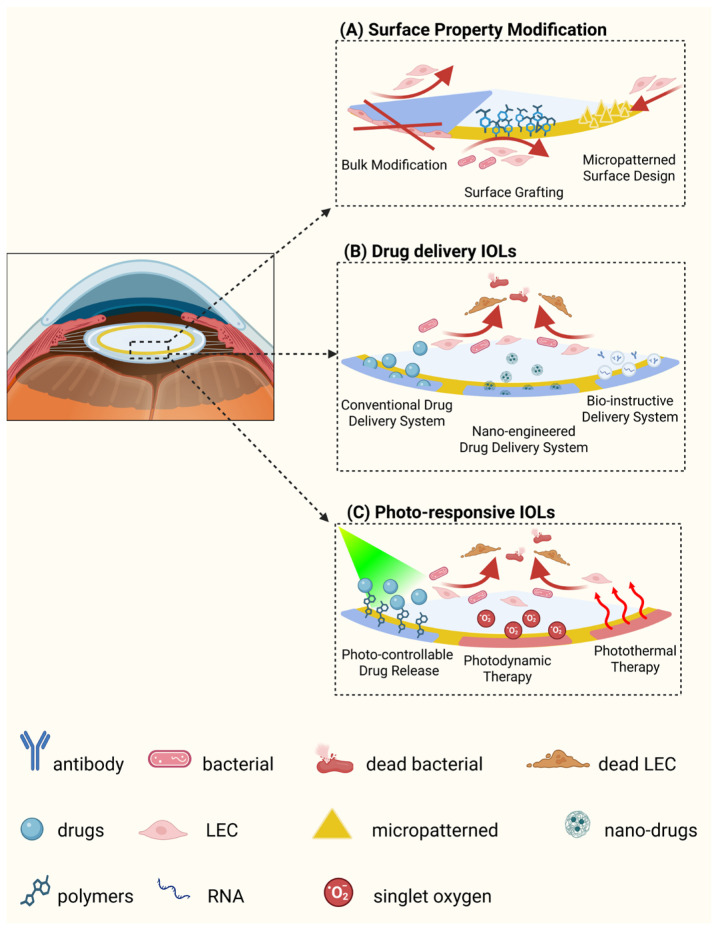
Schematic diagram of IOL modification strategies. (**A**) Surface modification strategies. (**B**) Drug delivery IOLs. (**C**) Photo-responsive IOLs. (Created in BioRender. Zeng, L. (2026) https://BioRender.com/kzr6b1a (accessed on 10 May 2026)).

Given the close association between postoperative complications and IOL material properties [34,55,56], extensive efforts have focused on IOL modification strategies. Approaches include surface modification [30,57,58,59,60,61] (Figure 1A), drug delivery IOLs [62,63,64,65] (Figure 1B), and photo-responsive IOLs [29,66,67,68] (Figure 1C). Recently, driven by the rapid interdisciplinary convergence of materials science, ophthalmology and pharmacology, combined modification strategies and multifunctional systems have gained attention to achieve synergistic effects [64,69,70]. Nevertheless, several challenges limit clinical translation. Most studies remain confined to in vitro or animal models [57,71] lacking robust clinical trials. Long-term safety and durability are rarely evaluated, and patient-specific applicability—particularly in eyes requiring premium IOLs—remains insufficiently addressed [72,73,74]. In addition, complex fabrication processes and increased manufacturing costs may hinder large-scale clinical adoption.

This review aims to systematically summarize the recent progress in IOL material modification for postoperative complication prophylaxis. Modification strategies are categorized into three major types—surface modification, drug delivery systems, and photo-responsive IOLs. By analyzing their mechanisms, advantages, and limitations, this review seeks to provide insights to accelerate the development of IOL modification [75] and bridge the gap between innovation and clinical translation.

## 2. Surface Property Modification

Surface property modification employs chemical and physical strategies to alter the surface property to prevent postoperative complications.

### 2.1. Bulk Modification

Clinical and experimental evidence indicates that hydrophobic IOLs are associated with a reduced incidence of PCO, which may be attributed to their favorable surface properties and biocompatibility [76,77,78]. Inspired by this mechanism, Wang et al. [79] synthesized a novel poly (hedral oligomeric silsesquioxane-co-methyl methacrylate) copolymer (allyl POSS-PMMA) via free radical polymerization to enhance human lens epithelial cell (HLEC) adhesion. In vitro, the resulting cell monolayer occupied the space between the IOL and posterior capsule, inhibiting PCO formation. However, several studies reported no significant PCO benefit of hydrophobic over hydrophilic IOLs, mainly due to greater adhesion [80,81,82,83]. To reduce LEC adhesion, Liu et al. [84] developed a novel IOL material through free radical copolymerization of ethylene glycol phenyl ether methacrylate (EGPEMA) and 2-(2-ethoxyethoxy) ethyl acrylate (EA). By adjusting monomer composition, researchers improved anti-adhesive performance to achieve LEC viability reduction while maintaining good biocompatibility. To take advantages of hydrophilic and hydrophobic properties, Hamedi et al. [85] synthesized amphiphilic copolymers of 2-hydroxyethyl methacrylate (HEMA) and 3-methacryloxypropyltris (trimethylsiloxy) silane (TRIS). Results revealed that copolymers with greater hydrophilicity could inhibit EMT with tissue-like roughness while copolymers with greater hydrophobicity promoted LEC adhesion. Therefore, new materials balancing hydrophilicity and hydrophobicity could improve PCO prevention without the use of therapeutics.

In summary, the prophylactic efficacy of IOL bulk modification relies on balancing surface hydrophobicity and hydrophilicity to modulate cellular behavior and inflammation [85,86]. Hydrophobic IOLs typically exhibit strong capsular adhesion, forming a passive physical barrier against short-term LECs but may fail to inhibit the molecular cascades of inflammation and even exacerbate EMT [87], which is relevant to other properties of hydrophobic IOLs such as roughness and stiffness. Conversely, hydrophilic IOLs confer remarkable anti-adhesive capabilities by forming a stable hydration layer that resists protein, LEC and bacterial adhesion. Furthermore, their tissue-like roughness and stiffness alleviate long-term postoperative inflammation and EMT in the downstream pathway [86]. Additionally, hydrophilic matrices facilitate active drug delivery systems, representing a revolution to active prophylactic strategy.

Achieving an optimal hydrophobic–hydrophilic balance is therefore paramount. Accumulating evidence from our collected literature suggests that an intermediate water contact angle (WCA) of 70–100° maximizes long-term PCO prevention by harmonizing necessary capsular adhesion with robust anti-biofouling properties [84,85,88]. Importantly, the specific hydrophobic-to-hydrophilic ratio required to reach this target WCA range is highly dependent on the inherent physicochemical properties of different materials. For instance, Hamedi et al. [85] considered the ratio of 1:3 could keep the balance between the suppression of LEC proliferation and the inhibition of EMT than the ratio of 1:1. However, Liu et al. [84] achieved excellent anti-adhesion and biosafety with a 7:3 ratio. Nevertheless, there is a paucity of research investigating bulk modification strategies for endophthalmitis prevention, prompting a recent shift toward multifunctional surface modification strategies.

### 2.2. Surface Grafting

Surface grafting immobilizes functional monomers or polymer chains onto the IOL surface to generate a stable and uniform coating, regulating key surface properties, including wettability and biocompatibility. Compared with bulk modification, chemical grafting enables localized multifunctionality, accurate control of surface chemistry, and sustained postoperative protection [89]. Heparin was among the earliest biomolecules applied for surface grafting. Multiple experimental studies demonstrated that heparin-modified IOLs could reduce postoperative inflammation and suppress cellular and protein adhesion in animal models [90,91]. The preventive effects of heparin-modified IOLs were further validated in clinical studies [92,93,94]. However, with increasing clinical experience, limitations such as restricted long-term efficacy have become evident [95,96]. Consequently, recent research has gradually shifted toward exploring alternative bioactive molecules for chemical grafting to achieve more durable and effective IOL surface modification. Bozukova et al. [97] grafted methoxy-poly(ethylene glycol) (mPEG) onto isocyanate-functionalized polyester (HEMA-co-methyl methacrylate (MMA)) hydrogel IOLs to create a protein- and cell-repellent surface. Results proved that PEG-modified surface completely inhibited LEC adhesion and proliferation by suppressing extracellular matrix-mediated cell attachment. To enhance the biocompatibility of hydrophobic acrylic IOLs, Tan et al. [59] grafted a hydrophilic poly methacryloyloxyethyl phosphorylcholine–methyl acrylic acid (p(MPC–MAA)) copolymer onto the surface via ammonia plasma treatment. The modified IOLs reduced protein adhesion and preserved anterior capsule transparency. However, PCO was not completely prevented, possibly due to excessive hydrophilicity, underscoring the need to balance hydrophilicity and bioadhesion in grafted layers. Ultraviolet (UV)-induced photografting polymerization has emerged as a simple, rapid, and cost-effective approach for surface modification. Huang et al. [88] first employed UV irradiation to graft 2-methacryloyloxyethyl phosphorylcholine (MPC) onto the different sites of hydrophobic acrylic IOLs. In vitro PCO model demonstrated that both the anterior surface-modified group and the control group had a lower LEC area than the posterior surface-modified group, indicating that grafting location should be tailored to the in vivo microenvironment. Surface chemical grafting produces uniform coatings minimizing delamination and ensuring long-term stability [98]. Nevertheless, specialized equipment and the resulting high production costs remain major barriers to large-scale clinical translation, highlighting the need for improved production strategies. Novel surface modification strategies have also been explored. Matsushima et al. [99] treated IOL surfaces with UV/ozone (O_3_) irradiation and argon (Ar) plasma to enhance IOL–posterior capsule adhesion. In rabbit eyes, both treatments markedly suppressed LEC proliferation, supporting the feasibility of reactive oxygen- and plasma-based modification. To further enhance the safety of modified IOLs, Babizhayev et al. [100] applied a platinum thin film onto IOL haptics via magnetron sputtering. The platinum-coated surface scavenged peroxide compounds and reactive oxygen species (ROS), thereby mitigating oxidative stress-induced damage and reducing postoperative inflammatory responses in vitro.

The feasibility of employing surface modification for endophthalmitis prophylaxis has likewise been explored. Huang et al. [101] improved the surface hydrophilicity of silicone IOLs by grafting MPC via air plasma treatment, resulting in reduced bacterial adhesion and sustained antibacterial activity after 18 h incubation. Similarly, the intrinsic anti-adhesive property of MPC was explored by Han et al. [102]. They fabricated zwitterionic MPC brushes on IOLs for improved biocompatibility and hydrophilicity. In vitro adhesion test showed that few LECs and bacteria were found on the IOL surface. An in vivo test further validated its prophylactic potential against postoperative complications. Choi et al. [103] developed an ionic polymer-coated elastic nanopillar array (NPA) using initiated chemical vapor deposition of a copolymer of 4-vinylbenzyl chloride (VBC) and 2-(dimethylamino)ethyl methacrylate (DMAEMA) to achieve strong antibacterial activity and preserve optical transmittance. In the antibacterial test, the coated NPA could elongate and rupture the bacterial cell membrane by elastic interactions to kill the bacteria.

Collectively, these emerging surface modification strategies highlight additional opportunities to modulate IOL surface properties for the prevention of postoperative complications.

### 2.3. Micropatterned Surface Design

LEC migration is driven by focal adhesion-mediated interactions at the IOL–tissue interface [104,105]. Accordingly, engineering microscale or nanoscale surface topographies on IOLs has emerged as an effective strategy for PCO prevention [105,106].

The Sharklet (SK) micropattern, inspired by shark skin morphology, has been confirmed to inhibit cellular adhesion. In 2015, Magin et al. [107] first developed an SK micropatterned protective membrane (PM) on IOLs to investigate its PCO-preventing effect. Effective LEC inhibition was observed, greater on protruding micropatterns than recessed micropatterns. Afterwards, Kramer et al. [108] confirmed the significant preventing effect of SK micropattern both in vitro and in vivo, although with mildly increased inflammation in rabbit eyes implanted with PMs, suggesting a potential risk of bacterial colonization on micropatterned surfaces. To enhance both PCO prevention and biosafety, Ellis et al. [109] incorporated an SK-patterned membrane into the ClearSight IOL, a hydrophobic IOL featuring a 360° square edge and an open-bag design to reinforce its barrier effect. In vivo experiments revealed that SK-patterned IOLs exhibited the lowest PCO severity and minimal Soemmerring’s ring formation, without abnormal inflammation or toxicity. To simplify the fabrication while preserving the optical performance, Seo et al. [61] employed femtosecond laser microfabrication to fabricate nanotextured micropatterns on poly (HEMA) substrates. Both in vitro and in vivo experiments demonstrated significantly reduced cell adhesion. Moreover, micropatterns with dimensions comparable to cell size were most effective, promoting LEC elongation and alignment, and elucidating a potential mechanism of cellular regulation.

Micropatterned IOL design establishes a physical barrier restricting cellular proliferation and migration, effectively reducing PCO formation. However, its role in preventing endophthalmitis remains controversial. Moreover, potential risks associated with residual fabrication byproducts, as well as increased manufacturing cost and processes, may limit clinical translation.

In summary, the three approaches of surface property modification reveal distinct advantages and limitations for each in mitigating postoperative complications. Bulk modification leverages intrinsic material wettability to deter PCO. However, reconciling opposing hydrophilic–hydrophobic dynamics is technically challenging, and its prophylactic potential against endophthalmitis is constrained by inadequate antimicrobial capacity. Surface modification remains a prevailing strategy offering highly versatile platforms for synergistic functionalization, enabling the precise tuning of physicochemical traits. This demonstrates its immense potential for integration with drug delivery systems and large-scale manufacturing in the future. However, a long-term in vivo effect and coating stability remain controversial. Micropatterned designs engineer robust topographical barriers that physically obstruct cellular migration. Nevertheless, broader clinical translation is impeded by its passive prevention efficacy against PCO and undetermined effects against endophthalmitis, alongside complex manufacturing logistics and the potential toxicity of residual fabrication byproducts.

## 3. IOL Drug Delivery Systems

To overcome the therapeutic limitations of surface modification, research has pivoted toward the development of IOL drug delivery systems (IOL-DDSs) designed to exert targeted antiproliferative and antimicrobial activity. Unlike surface modification that merely alters intrinsic physical or chemical properties, the functionalized IOLs offer enhanced clinical efficacy by integrating specific therapeutic agents against bacteria and LECs [110]. Furthermore, localized delivery via IOLs bypasses the systemic toxicity and high dosage requirements in systemic pharmacological prophylaxis [71]. Generally, these IOL-DDSs could be classified into three categories: conventional DDSs, nano-engineered DDSs (NDDSs) and bio-instructive delivery systems.

### 3.1. Conventional DDSs

Conventional DDSs primarily encompass matrix-based DDSs and drug-coated IOLs. Matrix-based DDSs integrate therapeutic agents directly into the IOL matrix via immersion or monomer modification, whereas drug-coated IOLs involve the fabrication of functionalized coating to encapsulate the agents [87]. Both approaches are instrumental in achieving sustained and autonomous drug release, ensuring a localized therapeutic effect post-implantation.

#### 3.1.1. Matrix-Based DDSs

Drug soaking remains a conventional technique for fabricating DDSs by immersing IOLs in pharmacological solutions to facilitate adsorption or reservoir formation [71,111]. Table 1 presents a summary of representative studies on drug-soaked IOLs. Early studies demonstrated that IOLs soaked in anti-inflammatory agents—including indomethacin [112], celecoxib (CXB) [113], and methotrexate (MTX) [114]—achieved superior cumulative release and extended duration compared to topical administration, effectively attenuating PCO. More recently, the scope has expanded to targeted molecular inhibitors such as erlotinib [115] and gefitinib [116] for targeted LEC elimination. For endophthalmitis prevention, both fluoroquinolone monotherapies (moxifloxacin (MXF) [117,118,119,120], gatifloxacin (GAT) [117,120,121] and levofloxacin (LEV) [121]) and dual-drug systems [122,123] have been engineered to maintain antibiotic concentrations above the minimum inhibitory concentration (MIC). Despite being cost-effective and clinically accessible, the clinical utility of drug-soaked IOLs is constrained by suboptimal release kinetics—specifically initial burst release and imprecise dose control—alongside potential solvent-induced alterations in the optical and physical properties.

To address the limitations of conventional soaking, supercritical fluid impregnation (SFI) has emerged as a sophisticated alternative for developing matrix-based DDSs. This approach exploits the gas-like diffusivity and liquid-like solvation capacity of supercritical CO_2_ (ScCO_2_) to infuse drugs into polymeric matrices under precisely controlled conditions [124]. SFI is increasingly recognized as a “green” and clinically compatible strategy as it minimizes burst release, preserves physicochemical integrity under mild conditions, and ensures the absence of toxic organic residues [125,126]. In early 2010s, Masmoudi et al. [127] and González-Chomón et al. [128] explored the feasibility of ScCO_2_-assisted antibiotic loading for endophthalmitis prophylaxis, which were unfortunately plagued by the foaming-induced loss of optical transparency. Subsequent optimizations by Bouledjouidja et al. [129,130] involving slow pressurization and depressurization successfully impregnated ciprofloxacin (CIP) and dexamethasone (DEX) sodium phosphate into IOL matrices. The produced IOLs maintained optical transparency and achieved sustained drug release. Building on this, Ongkasin et al. [131] loaded MTX on ScCO_2_-impregnated hydrophobic acrylic IOLs for PCO prophylaxis, effectively suppressing fibrotic responses and EMT in human capsular bag models. These advancements in SFI optimization provide valuable insights for the development of high-performance and clinically available IOL-DDSs.

Recent innovations seek to streamline fabrication while maximizing therapeutic efficacy. Hong et al. [65] developed a novel poly (glycidyl methacrylate-co-2-(2-ethoxyethoxy) ethyl acrylate) (PGE)-based IOL matrix that incorporates doxorubicin (DOX) directly into the lens matrix, achieving complete PCO prevention in vivo while maintaining excellent transparency and foldability. Furthermore, Li et al. [132] employed photocuring-based 3D printing to produce GAT-loaded IOLs that matched the optical and mechanical performance of commercial standards while providing sustained antimicrobial activity. With continued interdisciplinary integration, these advancements in matrix-based DDS design offer a promising pathway toward high-performance, customizable clinical IOLs.

#### 3.1.2. Drug-Coated IOLs

Coatings have emerged as a versatile platform for DDSs, offering high loading capacity and tunable surface functionalization [111]. Table 2 summarized the key studies on drug-coated IOLs.

In 2009, Liu et al. [133] first fabricated rapamycin (RAPA)-loaded poly (lactic-co-glycolic acid) (PLGA) coatings on polymethyl methacrylate (PMMA) IOLs via spray coating, demonstrating the potent inhibition of LEC proliferation and fibrin accumulation. To improve therapeutic efficacy, Kassumeh et al. [134] developed MTX coatings using similar materials. Despite significant antiproliferative effects in vitro, cell adhesion at the IOL edge was not completely inhibited. Subsequent research by Liu et al. [64] addressed this by grafting hydrophilic monomer MPC onto IOL surfaces to construct DOX-loaded polydopamine (PDA) coatings, which effectively suppressed PCO by enhancing surface hydrophilicity and inducing LEC apoptosis. To preserve optical performance, recent advancements have focused on the spatial optimization of coating architecture. Lu et al. [69] constructed a centrifugally concentric PLGA coating loaded with cyclosporin A (CsA), which maintained a thin central optic for visual clarity while providing a thick peripheral reservoir for LEC inhibition. Alternatively, restricting modification to the non-optical zone has proven effective. Accordingly, Zhang et al. [135] utilized ultrasonic spray coating to deposit bromfenac-loaded PLGA onto the plate haptics of IOLs, which preserved optical transparency while effectively suppressing fibrotic responses in vivo. For scalable manufacturing, Chen et al. [136] fabricated a thermoreversible agarose (Aga) coating through temperature-triggered process. This method enabled controlled drug release and maintained posterior capsule clarity, offering a low-cost, scalable approach with promising translational potential. In addition to conventional synthetic organic polymers, Wang et al. [137] first introduced a drug-loaded metal–polyphenolic network (MPN) coating on IOLs via self-assembly, which reduced postoperative inflammation and cell adhesion in vivo, though the initial burst release needed further optimization.

Drug-coated IOLs have also emerged as a viable strategy for endophthalmitis prophylaxis. Garty et al. [138] pioneered this application by developing a biocompatible poly-HEMA hydrogel coating on IOL haptics to achieve the sustained release of norfloxacin. Utilizing common and biosafe materials made the design highly amenable to industrial scale-up and clinical translation. To further optimize drug-loading capacity, Li et al. [139] loaded amikacin (AMK) on the surfaces of hydrophilic IOLs coated with zwitterionic poly(carboxylbetaine-co-dopamine methacrylamide) copolymers (pCBDA) and dopamine (DA). This synergistic coating not only enhanced anti-biofouling and antibacterial properties but also delivered an elevated drug payload with prolonged release kinetics. Nevertheless, compared to the extensive research on PCO, there remains a distinct paucity of studies dedicated to endophthalmitis prophylaxis.

Coatings constitute an effective and technically mature approach for fabricating IOL-DDSs, offering biocompatibility and large-scale manufacturing [111]. Nevertheless, the possible impact on optical performance and the management of initial burst release remain critical hurdles for clinical implementation.

### 3.2. Nano-Engineered Drug Delivery Systems

NDDSs leverage nanoparticles or nanofilms to achieve precise control over architectural thickness and tunable release kinetics. Compared to conventional DDSs, NDDSs offer a superior therapeutic window by enabling the sophisticated design of drug release profiles [110,140]. Table 3 summarizes the representative studies on IOLs with NDDSs.

Layer-by-layer (LbL) assembly, a paradigmatic strategy utilizing the electrostatic attraction between oppositely charged components to construct functional nanofilms or nanoparticles, remains a cornerstone of this field [141,142]. In this strategy, therapeutic agents could be integrated into multilayer films by directly embedding within the multilayer network or incorporating drug-loaded nanocarriers into LbL membranes. By precisely modulating parameters such as the number of layers, molecular composition, and cross-linking density, LbL deposition provides a robust platform for high-capacity drug loading and programmable, localized delivery tailored for the IOL surface. Manju et al. [143] first demonstrated the feasibility of LbL by constructing ampicillin-loaded nanofilms, achieving high reproducibility in drug elution. Parallel to LbL, hydrogel-based nanofilms represent another high-potential strategy due to their inherent hydrophilicity and biocompatibility [144]. Saraswathy et al. [75] synthesized amphiphilic siloxane nanogels via spin coating to carry DEX, sustaining therapeutic levels for 168 h. Similarly, PLGA/polycaprolactone (PCL) blends have been widely adopted as nanoporous drug reservoirs due to their biocompatibility and biodegradability [145,146]. Both Lamprogiannis et al. [147] and Karamitsos et al. [148] utilized these blends for spin-coated DEX nanofilms, with the latter further demonstrating supplementary ultraviolet-protective capabilities. With the technical maturity of LbL, Han et al. [60] incorporated DOX-loaded chitosan-tripolyphosphate (CHI-TPP) nanoparticles into heparin-based multilayers to create a pH-responsive system that significantly suppressed PCO. However, the effective inhibition of LEC migration required at least five nanolayers, a thickness that began to compromise optical transmittance. To balance sustained release with optical clarity, Huang et al. [63] developed hyaluronic acid (HA)/CHI multilayers that achieved sustained paclitaxel release while maintaining high transmittance and improving surface hydrophilicity. Further advancing the functionality of nanoparticles, Qin et al. [149] encapsulated DOX in polyaminoamide (PAMAM) to formulate cationic DOX@PAMAM nanoparticles via the LbL technique. The modified IOLs significantly prevented PCO in rabbit eyes through the synergistic antiproliferative effect of chemotherapy and autophagy induced by PAMAM.

**Table 3:** Representative studies on IOLs with NDDSs.

Reference	Year	IOL Material	Drug (Concentration)	Delivery Platform Material	Drug Loading Strategy	Experiment/Observation Period	Biological Evaluation
Manju et al. [143]	2010	PMMA	ampicillin (1 mg/mL)	PSS and PEI	LbL	drug release test: 7 d	achieve sustained drug release
Saraswathy et al. [75]	2016	hydrophobic acrylic	DEX (1 mg/mL)	siloxane nanogel	soaking	drug release test: 168 h	alleviate postoperative inflammation
Lamprogiannis et al. [147]	2018	Silicone	DEX (0.67 mg/sample, 0.5 mg/sample)	PLGA/PCL blend	encapsulation	drug release test: 10 w	form nanoporous structure with high encapsulation; achieve sustained clinical release
Karamitsos et al. [148]	2020	PMMA	DEX (74.10 μg/sample)	PLGA/PCL blend	encapsulation	drug release test: 8 w	achieve sustained drug release
Han et al. [60]	2019	foldable hydrophobic acrylic	DOX (0.05%)	heparin	LbL	HLECs: 72 h	inhibit cell adhesion, proliferation, and migration
						rabbit eyes: 10 w	significantly reduce PCO and Soemmerring’s ring formation
Huang et al. [63]	2021	PDMS	paclitaxel (NA)	HA and CHI	LbL	HLECs: 48 h and 72 h	inhibit cell proliferation
Qin et al. [149]	2021	NA	DOX (1.3 μmol/mL)	PAMAM and haparin	LbL	LECs: 24 h	inhibit HLEC proliferation
						rabbit eyes: 5 w	no pronounced PCO for 30 d
Vieira et al. [150]	2017	hydrophilic acrylic	MXF (5 mg/mL)	poly-HEMA	entrapment and soaking	microfluidic assay: 15 d	maintain effective antimicrobial levels for long-term endophthalmitis prevention
						antibacterial tests: 24 h	maintain MFX concentrations above MIC for *S. aureus* and *S. epidermidis* for 12 d
Pimenta et al. [151]	2017	hydrophilic polymethacrylate	MXF (5 mg/mL)	AMPS or SBMA	soaking	drug release test: 21 d	AMPS-modified samples show higher release profile than SBMA-modified samples
						antibacterial tests: 15 d	inhibit *S. aureus* and *S. epidermidis* for up to 12 d
Xiang et al. [152]	2021	hydrophobic acrylic	GS (1 mg/mL)	PDA	grafting	HLECs: 24 h	inhibit bacterial adhesion, decrease biofilm thickness, and inhibit HLEC adhesion

Abbreviations: 2-acrylamido-2-methylpropane sulfonic acid (AMPS), chitosan (CHI), dexamethasone (DEX), doxorubicin (DOX), gentamycin (GS), hyaluronic acid (HA), 2-hydroxyethyl methacrylate (HEMA), human lens epithelial cells (HLECs), layer-by-layer (LbL), lens epithelial cell (LEC), minimum inhibitory concentration (MIC), moxifloxacin (MXF), polyaminoamide (PAMAM), polycaprolactone (PCL), posterior capsule opacification (PCO), polydopamine (PDA), polydimethylsiloxane (PDMS), poly (ethylenimine) (PEI), poly (lactic-co-glycolic acid) (PLGA), polymethyl methacrylate (PMMA), poly (sodium 4-styrenesulfonate) (PSS), and [2-(methacryloyloxy)ethyl]dimethyl-(3-sulfopropyl) ammonium hydroxide (SBMA).

Beyond PCO prophylaxis, NDDSs have been tailored to combat endophthalmitis. Vieira et al. [150] and Pimenta et al. [151] utilized argon plasma-assisted grafting to immobilize MXF within superabsorbent nano-hydrogels, achieving a sustained 12-day antibiotic release without a significant initial burst effect. In a notable move toward multifunctional protection, Xiang et al. [152] designed asymmetric acrylic IOLs featuring a gentamicin (GA)-loaded anterior surface and an LEC-inhibiting posterior surface. This dual-action design highlights the potential of NDDSs to provide comprehensive postoperative protection.

Collectively, NDDSs represent the evolution of conventional DDSs, characterized by superior thickness control, preserved optical clarity, and more predictable release profiles. Although the management of initial burst release requires further refinement, NDDSs stand as a promising frontier for the next generation of multifunctional IOLs.

### 3.3. Bio-Instructive Delivery Systems

Distinct from conventional pharmacological agents, bio-instructive delivery systems are engineered to transport high-precision biomolecules, such as antibodies [153,154], nucleic acid therapeutics [153,155] and enzymes [156], to achieve targeted therapy with enhanced biosafety profiles. Table 4 presents the representative studies on IOLs with bio-instructive delivery systems.

Given the pivotal role of transforming growth factor β2 (TGF-β2) in driving the EMT of LECs, Sun et al. [157] utilized LbL assembly to fabricate anti-TGF-β2 antibody multilayers on IOL surfaces while preserving their immunological activity. Although these antibody-functionalized IOLs significantly attenuated LEC migration and EMT, the suppression of cell adhesion remained transient, with a negligible impact on proliferation. Advancing targeted bioactive molecules toward gene therapy, Wang et al. [158] engineered a non-viral gene-delivery coating by integrating poly (ethylenimine) (PEI)–PEG electrostatic complexation with LbL deposition to deliver platelet-derived growth factor receptor-α (PDGFR-α) short hairpin RNA (shRNA). In rabbit models, these gene-silencing IOLs significantly reduced early PCO incidence without adverse effects on surrounding ocular tissues, highlighting the precision of RNA interference therapy in PCO prophylaxis. Further leveraging the advantages of homologous targeting and high biocompatibility of exosome, Zhu et al. [159] immobilized DOX-loaded exosomes onto IOL surfaces. The stability was validated by the similar release behavior to the profile of DOX in the release buffer. These bio-instructive carriers enhanced bioavailability and exploited targeted delivery while eliminating burst release. To circumvent the collateral toxicity associated with conventional chemotherapy, Jia et al. [160] developed a cascade catalytic system utilizing natural enzymes immobilized on mesoporous silica nanoparticles, which were then anchored to IOLs via LbL self-assembly. The modified IOLs exhibited excellent biocompatibility and effective LEC elimination in vitro and in vivo by inducing glucose-triggered ROS-mediated apoptosis. Without the need for cytotoxic drugs or external energy, this design minimizes systemic adverse effects and establishes a novel paradigm for “drug-free” IOL modification.

Bio-instructive delivery systems represent a sophisticated frontier for enhancing therapeutic outcomes tailored to the high sensitivity of ocular microenvironment [161,162]. Nevertheless, no nucleic acid- or antibody-modified IOLs have received FDA or EMA approval. Currently, their clinical translation is severely restricted by the stringent requirements. Future studies are expected to investigate targeting bioactive agents with long-term stability and scalable production while maintaining excellent biosafety.

In summary, the three primary IOL-DDS strategies present both advantages and limitations. Conventional DDSs provide high loading capacity and maturity in the processing technique [163]. However, their clinical implementation is frequently hindered by a pronounced initial burst release and the potential to compromise the optical transparency of the IOL. Moreover, the reliance of traditional therapeutic agents such as DEX may decrease the prevention efficacy due to their undesirable diffusion and increasing risk of complications [164]. In contrast, NDDSs provide precise and sustained drug release with minimal impact on optical quality [146]. Despite these advancements, it remains a persistent challenge to achieve on-demand prevention. Bio-instructive delivery systems achieve targeted elimination of LECs with excellent biosafety [165,166], perfectly tailored to the highly sensitive ocular microenvironment. Nevertheless, their broader clinical translation is currently impeded by undetermined long-term in vivo stability and the inherent complexities of scalable production. Future studies focusing on refined release kinetics by applying mathematical models [167] and the use of bio-integrated materials [87] are essential to achieving the full potential of drug-loaded IOLs.

## 4. Photo-Responsive IOLs

The aforementioned prophylactic strategies are predominantly passive and autonomous, lacking capacity for precise, self-regulated modulation to meet individualized patient requirements. To address these limitations, a growing number of studies have focused on the integration of photo-responsive materials into IOL modification to facilitate controllable, on-demand prophylaxis against postoperative complications, marking the rise of personalized precision medicine [87,111]. Prominent strategies in this field include photo-controllable drug release, photodynamic therapy (PDT), and photothermal therapy (PTT).

### 4.1. Photo-Controllable Drug Release IOLs

Photo-controlled drug release IOLs integrate photo-responsive materials with IOLs to create “smart” drug delivery systems capable of on-demand and spatially controlled drug release after implantation. Unlike autonomous systems, these IOLs allow for selectively suppressing LEC proliferation while minimizing off-target ocular toxicity [168].

In a seminal study, Kim et al. [169] synthesized coumarin-functionalized acrylic IOLs, with the antiproliferative agents 5-fluorouracil (5-FU) and chlorambucil covalently linked via coumarin-derived photosensitive linkers. Under two-photon absorption irradiation (532 nm), the modified IOL achieved noninvasive, light-triggered drug release and the effective inhibition of cell proliferation without dark toxicity, thereby offering a viable path for personalized PCO prophylaxis. To improve processability, Sinkel et al. [170] developed low-molecular-weight coumarin-based polymers to serve as photo-responsive drug depots. Triggered by single-photon irradiation, the modified IOL achieved controllable, light-dependent drug release. Based on the published studies, Xia et al. [171] and Hu et al. [62] incorporated coumarin methacrylate (CMA) into IOLs coatings or matrices, enabling rapid, controllable 5-FU release upon irradiation. These photo-responsive IOLs enabled rapid and tunable 5-FU release upon irradiation, significantly reducing cellular adhesion and PCO progression in vivo.

While photo-controllable IOLs represent a major advancement toward active, on-demand therapeutic intervention, several hurdles remain. Challenges related to irradiation safety, limited light penetration, unavoidable drug toxicity, and the lack of long-term clinical data currently constrain their clinical translation.

### 4.2. Photodynamic IOLs

Photodynamic therapy (PDT) has emerged as a compelling strategy for preventing postoperative complications due to its superior spatiotemporal controllability and favorable biosafety. Compared to photo-responsive drug release systems depending on the elution of potentially toxic pharmacological agents, PDT could selectively eliminate LECs and pathogens by singlet oxygen (^1^O_2_) generated by photosensitizers under specific irradiation [111].

Initial efforts utilized PLGA as a carrier for photosensitizer loading for its excellent biodegradability [172]. Building on this, Zhang et al. [173] reported the first photodynamic IOL by encapsulating indocyanine green (ICG) within a PLGA matrix for PCO prevention. Although this system achieved light-dependent LEC ablation in vivo, its clinical translation was constrained by delayed visual recovery and residual ICG pigments. To enhance photodynamic efficiency, research shifted toward Chlorin e6 (Ce6), a potent and high-yield ROS generator [174]. Tang et al. [29] grafted Ce6 onto α-cyclodextrin (α-CD) and immobilized it on poly(poly(ethylene glycol) methacrylate) (PPEGMA) brush layers. The modified IOLs not only suppressed LEC adhesion via enhanced hydrophilicity but also achieved the complete elimination of adherent cells under 660 nm irradiation. To refine the structural design for clinical relevance, Lu et al. [66] developed an annular Ce6@PLGA coating. By modifying the lens periphery to induce ^1^O_2_-mediated apoptosis, this design significantly attenuated the formation of Elschnig’s pearls and Soemmerring’s ring while preserving central optical quality. The frontier of PCO prevention involves the integration of multimodal synergistic. Qie et al. [175] developed a PDA-based Ce6 coating that utilized the photothermal conversion of PDA to enhance photodynamic activity, achieving rapid LEC elimination and sustained suppression for four weeks. Similarly, Fang et al. [176] designed an ICG-loaded PDA coating on IOLs (IP-IOLs) to prevent PCO. Under near-infrared irradiation, the IP-IOLs induced oxidative stress and localized hyperthermia, ensuring effective LEC necrosis and maintaining central optical clarity for up to 28 days in vivo.

In addition to PCO, PDT has demonstrated significant promise in endophthalmitis prophylaxis. In 2009, Parsons et al. [177] first fabricated localized photodynamic IOL surfaces via porphyrin impregnation, demonstrating inherent antibacterial activity that could be markedly accelerated under intense light. To simplify activation conditions, McCoy et al. [178] utilized MMA to localize high-concentration tetracationic porphyrin on IOL surfaces, which generated ^1^O_2_ exceeding clinical requirements. Notably, the catalytic reaction enabled a sustained antibacterial effect rather than photosensitizer consumption. The resulting inhibition of bacterial adhesion and the ability to filter short-wavelength light underscore its potential as a multifunctional clinical candidate.

Collectively, PDT-based IOL modification offers a paradigm of precise controllability, potent cellular eradication, and robust biosafety. As material synthesis and fabrication strategies continue to mature, PDT holds substantial promise for clinical translation [179]. Nevertheless, rigorous longitudinal evaluations of safety and efficacy within the complex physiological environment of the eye remain essential for future implementation.

### 4.3. Photothermal IOLs

Photothermal therapy (PTT) incorporates photothermal agents into IOLs to generate localized hyperthermia upon near-infrared (NIR) irradiation, inducing cell death and preventing PCO [180]. Similar to PDT, the development of PTT-modified IOLs necessitates a balance between high photothermal conversion efficiency and robust biocompatibility.

Early studies tended to combine PTT with drug delivery to enhance therapeutic potency. Leveraging the superior drug-loading capacity and photothermal properties of black phosphorus (BP) [181], Mao et al. [182] developed DOX-loaded, BP-modified IOLs that achieved spatiotemporally controlled drug release and selective LEC elimination under NIR triggers. Afterwards, Ye et al. [68] utilized Ti_3_C_2_ MXene and RAPA via spin coating to enable region-confined LEC inhibition. To further mitigate potential drug-related toxicity, Qin et al. [67] introduced a low-power, thermosensitive IOL design that maintained capsular clarity for 28 days through precisely controlled drug release.

To circumvent the chronic risks of pharmacological toxicity, subsequent studies have pivoted toward drug-free photothermal strategies. Gold nanoparticles (AuNPs) are widely utilized for their intense light absorption and localized heating effects [183]. Hong et al. [184] integrated AuNPs into metal–organic frameworks (AuNPs@MIL-101-NH2 (MIL)-PGE) to induce cell death through a multifaceted mechanism of starvation, ferroptosis, and hyperthermia. However, attenuation of AuNP activity due to protein adsorption in the ocular environment raised concerns on long-term efficacy. To improve spatial selectivity and safety, Liu et al. [185] restrictively modified the non-optical region of IOLs with AuNPs, enabling peripheral temperature elevation to eliminate LECs while preserving optical center transparency. Reduced graphene oxide (rGO), offering higher NIR absorption efficiency and lower cost, has also emerged as a promising photothermal material [186]. Zhang et al. [187] demonstrated that rGO-modified IOLs effectively suppressed LEC migration and proliferation under NIR irradiation, resulting in reduced PCO incidence. Moving toward even safer clinical profiles, Lin et al. [188] developed a PDA/polyvinyl alcohol hydrogel ring that utilizes prolonged, low-intensity heating to inhibit LEC adhesion and migration, significantly reducing the risk of collateral thermal damage. The feasibility to build multifunctional adjustable IOLs based on PTT has been explored by Chen et al. [189], who combined thermosensitive triblock-polymer pluronic F127 diacrylate (F127DA), hyaluronic acid methacrylate (HAMA), BP, tannic acid (TA), and silver nanoparticles (Ag NPs) to fabricate a thermal-/photo-cross-linkable hydrogel. After conformational transition triggered by NIR, the IOLs displayed significant antibacterial and anti-inflammatory effects in vitro and vivo. To leverage the complementary advantages of different strategies, Zhang et al. [190] incorporated enzyme-based catalytic system with photothermal effect by modifying IOLs with AuNPs anchored onto metal–organic framework. The cascade catalytic system on modified IOLs produced ROS and consumed glucose and O_2_, which inhibited proliferation and migration of LECs. Simultaneously, NIR-triggered PTT enabled controllable PCO prevention. This multi-modal approach achieves safe, high-efficiency PCO prophylaxis, establishing a future research paradigm centered on the synergistic integration of biomimetic materials and advanced therapeutic strategies.

PTT-modified IOLs provide a highly controllable and effective means of LEC elimination [87]. Nevertheless, the path to clinical translation requires the further refinement of photothermal materials and irradiation parameters to ensure long-term stability and minimize thermal risks within the delicate ocular environment [111].

In summary, photo-responsive IOLs leverage light as a precise external trigger to enable controllable, on-demand prophylaxis against postoperative complications. As the novel interdisciplinary frontier integrating biomaterials and ophthalmology, this research direction holds profound potential for clinical translation. Photo-controllable drug release systems provide the advantage of active, on-demand therapeutic intervention. However, their clinical translation is constrained by the limited pharmacological versatility of 5-FU as well as the single prevention against PCO. PDT offers a paradigm of precise spatiotemporal controllability and potent cellular eradication with robust biosafety. Notably, its prophylactic efficacy against both PCO and endophthalmitis has also been explored and proven, demonstrating its versatile potential. Nonetheless, its implementation necessitates rigorous longitudinal evaluations of safety within the complex physiological ocular microenvironment. PTT also provides a highly controllable and effective means for LEC elimination, but its translational path requires the meticulous refinement of photothermal agents and irradiation parameters to strictly mitigate the risk of collateral thermal damage to delicate ocular tissues. Moreover, prevention against endophthalmitis should be one of its future directions. Collectively, while these novel interdisciplinary platforms hold profound translational potential for positive and on-demand postoperative prophylaxis, further rigorous refinement and expansion to endophthalmitis prevention are needed before widespread implementation.

## 5. Conclusions and Perspectives

IOL modification has demonstrated promising and versatile prevention for postoperative complications. Collectively, these strategies reflect an evolution from physical barriers to sophisticated chemical and biological interventions; from single-material optimization to multifunctional system integration; and from passive prevention to active, controllable and “smart” interventions, underscoring the growing potential of material-driven strategies in improving long-term surgical outcomes.

Despite these technological strides, current studies are still confined to in vitro or animal models due to concerns regarding the long-term stability and safety of modified IOLs in the physiological ocular environment. Technical challenges, such as the imprecise control of drug dosage, the potential for drug degradation, complex fabrication protocols, and the associated high manufacturing costs, continue to limit the feasibility of these next-generation IOLs in standard surgical practice.

The future development lies in bridging the gap between bioengineering innovation and clinical utility. First, as a long-term ocular implant, rigorous and longitudinal clinical evaluations should be complete to ensure no compromise of optical clarity or induced complications like chronic inflammation. Given the progressive nature of postoperative complications, there is a significant clinical need for degradable drug-eluting IOLs that provide controllable and long-term release without requiring secondary interventions. Moreover, synergistic approaches combining diverse modification strategies (e.g., integrating surface modification with drug delivery system) should be adopted, which may help achieve comprehensive prevention while reducing reliance on high drug doses. Last but not least, to ensure accessibility and reduce costs, manufacturing processes must be simplified and standardized. With the gradual advancement, the next generation of modified IOLs will evolve from mere refractive implants into proactive therapeutic devices, significantly improving long-term visual prognosis and patient satisfaction after cataract surgery.

## Figures and Tables

**Table 1:** Summary of representative studies on drug-soaked IOLs.

Reference	Year	IOL Material	Drug (Concentration)	Experiment/Observation Period	Biological Evaluation
Nishi et al. [112]	1995	PMMA	indomethacin (0.1% or 1.0%)	rabbit lens epithelial cells: 6–12 m	inhibit LEC proliferation and accumulation
				rabbit eyes: 4 w	lower PCO incidence
Davis et al. [113]	2012	hydrophobic and hydrophilic acrylic	CXB (20 μM and 300 mM)	HLECs: 28 d	complete LEC inhibition; no EMT
				canine lens capsular bag model: 28 d	reduce cell infiltration; lower PCO incidence
Wertheimer et al. [114]	2017	hydrophobic and hydrophilic acrylic	CA (1.6 ± 0.9 nM), disulfiram (359 ± 33 nM), MTX (98.0 ± 29.7 nM), RAPA (70.2 ± 14.0 pM), RA (1.1 ± 0.12 nM)	HLECs: 72 h	inhibit cell proliferation in MTX groups and CA groups
				human capsular bag model: 57–65 d (MTX), 21.7 d (CA), 10.7–11.3 d (RE), 11–11.7 d (RA), 9–10 d (control)	prolong confluence and inhibit PCO in MTX-loaded IOLs
Wertheimer et al. [115]	2018	hydrophilic acrylic, hydrophobic acrylic and hydrophilic acrylic with a hydrophobic surface	Erlotinib (unsaturated solution: 30 μm; supersaturated solution: NA)	HLECs: 72 h	inhibit LEC migration and proliferation
				human capsular bag model: 8 d (24 h treatment), 11.8 d (72 h treatment), 5.9 d (control)	prolong the time to total cell coverage of the capsular bag
Kassumeh et al. [116]	2021	hydrophobic and hydrophilic acrylic	Gefitinib (50 μM)	HLECs: 72 h	inhibit cell proliferation and migration; lower fibronectin; reduce fibrosis
				human capsular bag mode: 6.3 d (experiment), 13.3 d (control)	prolong time to confluence; lower PCO incidence
Kleinmann et al. [117]	2006	hydrophilic acrylic	GAT (NA), MXF(NA)	rabbit eyes: 6 h	maintain antimicrobial levels; lower endophthalmitis incidence
Shimizu et al. [121]	2006	hydrogel, PMMA, hydrophilic acrylic	LEV (0.5%), GAT (0.3%)	antibacterial tests: 72 h	reduce bacterial adhesion and inflammation
			GAT (0.5%)	rabbit eyes: 72 h	lower incidence of corneal opacity and conjunctival hyperemia
Lipnitzki et al. [120]	2013	hydrophilic acrylic	GAT (0.3%), MXF (0.5%), prednisolone acetate (1%)	rabbit eyes: 10 h	time-dependent loading; sustained drug release
Lipnitzki et al. [118]	2014	hydrophilic acrylic	MXF (5 mg/mL)	rabbit eyes: 10 h	prevent endophthalmitis in combination of soaking and intracameral injection
Yovel et al. [119]	2016	hydrophilic acrylic	MXF (5 mg/mL)	rabbit eyes: 24 h	reduce endophthalmitis and hypopyon incidence
Topete A et al. [122]	2019	acrylic CI26Y material	MXF (5 mg/mL), KTL (5 mg/mL)	antibacterial tests: 26 d	inhibit bacterial activity
Topete A et al. [71]	2021	acrylic CI26Y material	MXF (2.56 mM), DFN (1.76 mM)	antibacterial tests: 14 d	inhibit bacterial activity

Abbreviations: caffeic acid phenethyl ester (CA), celecoxib (CXB), diclofenac (DFN), epithelial–mesenchymal transition (EMT), gatifloxacin (GAT), human lens epithelial cells (HLECs), ketorolac (KTL), levofloxacin (LEV), moxifloxacin (MXF), methotrexate (MTX), posterior capsule opacification (PCO), polymethyl methacrylate (PMMA), rapamycin (RAPA), and retinoic acid (RA).

**Table 2:** Key studies on drug-coated IOLs.

Reference	Year	IOL Material	Drug (Concentration)	Delivery Platform Material	Drug Loading Strategy	Experiment/Observation Period	Biological Evaluation
Liu et al. [133]	2009	PMMA	RAPA (40 μg/sample)	PLGA	spray coating	rabbit eyes: 14 d	inhibit LEC proliferation; lower PCO incidence
Kassumeh et al. [134]	2018	hydrophobic acrylic	MTX (NA)	PLGA	spray coating	human capsular bag model: 14 d	inhibit LEC growth and migration; delay PCO formation
Liu et al. [64]	2021	foldable hydrophobic acrylic (FV-60A)	DOX (0.5 mg/mL)	PDA-2-MPC	soaking	HLECs: 72 h	achieve sustained release; induce HLEC apoptosis
						rabbit eyes: 6 w	achieve complete inhibition of PCO formation
Lu et al. [69]	2022	foldable hydrophobic acrylic (Pho)	CsA (5 mg/mL)	PLGA	spin coating	HLECs: 72 h	induce autophagy-mediated cell death; inhibit LEC proliferation
						rabbit eyes: 1 m	lower PCO incidence
Zhang et al. [135]	2022	acrylic	BF (0.1%)	PLGA	spray coating	HLECs: 72 h	suppress EMT-related phenotypic changes; reduce LEC migration and proliferation
						rabbit eyes: 2 m	reduce the PCO severity
Chen et al. [136]	2022	foldable hydrophobic acrylic (Pho)	DOX (400 μg/mL)	Aga	soaking	HLECs: 72 h	efficiently eliminate LECs
						rabbit eyes: 21 d	lower PCO scores
Wang et al. [137]	2024	hydrophobic acrylic	AZD0364 (5 nmol/L), PTE (25 μg/mL)	metal–polyphenolic network	self-assembly	HLECs: 24 h	inhibit cell migration and adhesion
						rabbit eyes: 30 d	prevent inflammation and PCO
Garty et al. [138]	2011	PhacoFLEX II, STAAR Elastic Lens, Foldable Silicone Multi-piece Lens	norfloxacin (1% (wt/vol))	poly-HEMA hydrogel	sonication and mild heat	antibacterial tests: 24 h	all S. epidermidis died within 24 h in the norfloxacin-loaded polymer device
						rabbit eyes: 32 d	prevent endophthalmitis for 4 weeks
Li et al. [139]	2023	hydrophobic acrylic	AMK (2 mg/mL)	pCBDA and DA	soaking	antibacterial tests: 24 h	inhibit cell migration and adhesion
						Sprague Dawley rat subcutaneous infection models: 3 d	no bacteria adhesion on the coated IOL

Abbreviations: agarose (Aga), bromfenac (BF), cyclosporine A (CsA), dopamine (DA), doxorubicin (DOX), 2-hydroxyethyl methacrylate (HEMA), human lens epithelial cells (HLECs), lens epithelial cell (LEC), 2-methacryloyloxyethyl phosphorylcholine (MPC), methotrexate (MTX), poly(carboxylbetaine-co-dopamine methacrylamide) copolymers (pCBDA), posterior capsule opacification (PCO), polydopamine (PDA), poly (lactic-co-glycolic acid) (PLGA), polymethyl methacrylate (PMMA), pterostilbene (PTE), and rapamycin (RAPA).

**Table 4:** Representative studies on IOLs with bio-instructive delivery systems.

Reference	Year	IOL Material	Drug (Concentration)	Delivery Platform Material	Drug Loading Strategy	Experiment/Observation Period	Biological Evaluation
Sun et al. [157]	2014	hydrophobic acrylic	anti-TGF-β2 antibody (50 μg/mL)	PEI and PLL	LbL	LECs: 48 h	inhibit LEC migration and EMT; transiently inhibit adhesion; no inhibitory effect on proliferation
Wang et al. [158]	2023	foldable hydrophobic acrylic	PDGFR-α shRNA (NA)	PEI–g–PEG	LbL	HLECs: 84 h	interfere with EMT; inhibit cell migration and PDGFR-α expression
						rabbit eyes: 2 w	lower PCO incidence
Zhu et al. [159]	2022	foldable hydrophobic acrylic (Pho)	DOX (optimal: 11.7 μg/mL)	LEC exosome	electroporation	HLECs: 10 h	achieve exosome-mediated homologous targeting drug delivery; inhibit HLEC proliferation
						rabbit eyes: 27 d	lower PCO incidence
Jia et al. [160]	2025	foldable hydrophobic acrylic	GOx (NA), HRP (NA)	mesoporous silica nanoparticles	LbL	HLECs: 72 h	induce cell apoptosis; lower cell viability
						rabbit eyes: 28 d	lower PCO incidence

Abbreviations: doxorubicin (DOX), epithelial–mesenchymal transition (EMT), glucose oxidase (GOx), human lens epithelial cells (HLECs), horseradish peroxidase (HRP), layer-by-layer (LbL), lens epithelial cell (LEC), posterior capsule opacification (PCO), platelet-derived growth factor receptor-α (PDGFR-α), poly (ethylenimine) (PEI), and poly(ethylene imine)-graft-poly(ethylene glycol) (PEI–g–PEG).

## Data Availability

No new data were created or analyzed in this study.
